# A Associação de Pressão Arterial Definida pelas Diretrizes ACC/AHA de 2017 e Risco de Doença Cardiovascular para Pessoas de Meia-Idade e Idosas na China: Um Estudo de Coorte

**DOI:** 10.36660/abc.20230785

**Published:** 2024-08-06

**Authors:** Qingyang Lu, Haijing Xie, Xuefeng Gao

**Affiliations:** 1 Xicheng District Guangwai Hospital Beijing China Xicheng District Guangwai Hospital - Cardiovascular Medicine, Beijing – China; 2 Chongming Hospital Affiliated to Shanghai University of Medicine and Health Sciences Shanghai China Chongming Hospital Affiliated to Shanghai University of Medicine and Health Sciences - Oncology, Shanghai – China; 3 Academician of the World Academy of Productivity Sciences Changchun China Academician of the World Academy of Productivity Sciences, Changchun – China

**Keywords:** Doenças Cardiovasculares, Hipertensão, Pessoa de Meia-Idade, Idoso

## Abstract

**Fundamento:**

A doença cardiovascular (DCV) é uma série de doenças que afetam o coração ou os vasos sanguíneos.

**Objetivos:**

Avaliar a relação entre os níveis de pressão arterial (PA) definidos pelo American College of Cardiology/American Heart Association (ACC/AHA) de 2017 diretriz e risco de DCV/doença cardiovascular aterosclerótica (DCVA) para pessoas de meia-idade e idosos na China.

**Métodos:**

Um total de 6.644 pessoas de meia-idade e idosas do Estudo Longitudinal de Saúde e Aposentadoria da China CHARLS (*China Health and Retirement Longitudinal Study*) foram finalmente incluídas. De acordo com a diretriz ACC/AHA de 2017, todos os indivíduos foram divididos em quatro grupos: PA normal, PA elevada, hipertensão estágio 1 e hipertensão estágio 2. O desfecho deste estudo foi considerado o risco de DCV e DCVA. Modelos de regressão COX univariados e multivariados foram adotados para examinar a relação da classificação de PA ACC/AHA de 2017 com o risco de DCV. Modelos de regressão logística univariada e multivariada foram utilizados para investigar a associação entre os níveis de PA e o risco de DCVA. Foram realizadas análises de subgrupos baseadas em idade, sexo e uso de medicamentos anti-hipertensivos. P<0,05 foi aceito como estatisticamente significativo.

**Resultados:**

Após ajustar todas as covariáveis, em comparação com pacientes de meia-idade e idosos com PA normal, descobrimos que pacientes com hipertensão estágio 1/2 estavam associados a um maior risco de DCV, separadamente. Simultaneamente, também observamos uma associação positiva entre indivíduos com PA elevada, hipertensão estágio 1, hipertensão estágio 2 e maior risco de DCVA no modelo totalmente ajustado. O resultado das análises de subgrupos indicou que a relação entre hipertensão estágio 1/2 e DCV/DCVA alta foi robusta em diferentes idades e sexos, e participantes sem uso de medicamentos anti-hipertensivos.

**Conclusão:**

A classificação da PA de acordo com as diretrizes de PA da ACC/AHA de 2017 pode ser aplicada à população chinesa.

## Graphical abstract

**Figura Central:**
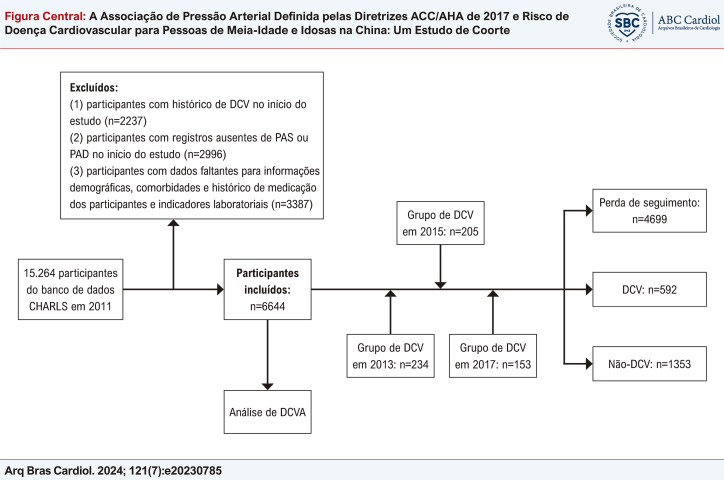
: A Associação de Pressão Arterial Definida pelas Diretrizes ACC/AHA de 2017 e Risco de Doença Cardiovascular para Pessoas de Meia-Idade e Idosas na China: Um Estudo de Coorte Fluxograma da população selecionada. DCV: doença cardiovascular; PAS: pressão arterial sistólica; PAD: pressão arterial diastólica.

## Introdução

A doença cardiovascular (DCV) é uma série de doenças que afetam o coração ou os vasos sanguíneos.^1^ Atualmente é reconhecida como a principal causa de morte em todo o mundo e é responsável por mais de 40% das mortes na China.^2^ Na China, a prevalência de DCV ainda está a aumentar com o desenvolvimento socioeconómico, o envelhecimento da população e as mudanças no estilo de vida.^3,4^ Estima-se que a prevalência de DCV tenha se duplicado desde 1990, atingindo quase 94 milhões em 2016, o que impôs um fardo aos custos de saúde e económicos.^2^ Portanto, compreender o impacto dos fatores de risco de DCV é fundamental para otimizar as medidas de prevenção de DCV.

É do conhecimento comum que a hipertensão está significativamente associada ao risco de DCV.^5^ Evidências mostram que pessoas de meia-idade e idosos têm maior probabilidade de sofrer de hipertensão.^6^ O manejo eficaz da hipertensão desempenha um papel importante na prevenção da prevalência de DCV em pessoas de meia-idade e idosos. Atualmente, a pressão arterial sistólica (PAS) ≥140 mmHg e/ou pressão arterial diastólica (PAD) ≥90 mmHg é comumente usada para definir hipertensão na população chinesa com base nas diretrizes de pressão arterial (PA) da CHL (*Chinese Hypertension League*) de 2018.^7^ No entanto, em 2017, o American College of Cardiology/American Heart Association (ACC/AHA) divulgou uma diretriz atualizada sobre novos critérios diagnósticos para hipertensão:^8^ hipertensão estágio 1 foi definida como PAS com 130-139 mmHg ou PAD com 80-89 mmHg; hipertensão estágio 2 foi definida como PAS≥140 mmHg ou PAD≥90 mmHg. As diretrizes da ACC/AHA podem superestimar a prevalência e o número de pacientes com hipertensão. Ainda não está claro se as diretrizes ACC/AHA de 2017 se aplicam à população chinesa. Vários estudos investigaram a associação entre hipertensão definida pelas diretrizes ACC/AHA de 2017 e risco de DCV entre a população chinesa.^9-11^ Um estudo epidemiológico realizado entre adultos com idade entre 35 e 49 anos em áreas rurais, na China, mostrou que a hipertensão em estágio 1 definida pelas diretrizes ACC/AHA de 2017 estava associada a um maior risco de acidente vascular cerebral.^11^ No estudo de Qi Y, et al., eles relataram que a hipertensão estágio 1 do ACC/AHA de 2017 estava relacionada ao risco cardiovascular entre adultos chineses jovens e de meia-idade (35-59 anos), mas não naqueles ≥60 anos de idade.^9^ Além disso, Xie YX, et al., também apontaram que o controle da PA em pacientes chineses idosos (≥60 anos) com hipertensão estágio 1 pode ajudar a reduzir o risco de DCV.^10^ Claramente, os resultados dos estudos sobre a relação entre a hipertensão sob os novos critérios e o risco de DCV na população chinesa ainda eram controversos até agora.

Aqui, o presente estudo teve como objetivo avaliar a relação entre a classificação de PA ACC/AHA de 2017 e o risco de DCV para pessoas de meia-idade e idosos na China com base no banco de dados do *China Health and Retirement Longitudinal Study* CHARLS. Além disso, também exploramos ainda mais a relação entre a classificação de PA ACC/AHA de 2017 e o risco de doença cardiovascular aterosclerótica (DCVA).

## Métodos

### População do estudo

Todos os dados deste estudo foram derivados do banco de dados CHARLS. O CHARLS adotou uma estratégia de amostragem em vários estágios cobrindo 28 províncias, 150 condados e 450 aldeias/comunidades urbanas, que coletou informações dos sujeitos sobre informações pessoais, família, estado de saúde, medição física, utilização de serviços médicos e seguros de saúde, trabalho, aposentadoria e pensões, renda, consumo, ativos e informações da comunidade.^12^ Os dados de base do CHARLS foram coletados em 2011, onda 2 em 2013, onda 3 em 2015 e onda 4 em 2018.^13^ O CHARLS foi aprovado pelo Comitê de Revisão Institucional da Universidade de Pequim. Todos os participantes receberam consentimento informado por escrito.

Para este estudo de coorte, selecionamos participantes do banco de dados CHARLS em 2011 (n=15.264). Excluímos alguns indivíduos que atendiam aos seguintes critérios: (1) participantes com histórico de DCV no início do estudo (n=2.237); (2) participantes com registros ausentes de PAS ou PAD no início do estudo (n=2.996); (3) participantes com dados faltantes sobre informações demográficas, comorbidades, histórico de medicação e indicadores laboratoriais dos participantes (n=3.387). Um total de 6.644 adultos elegíveis com idade ≥45 anos foram incluídos neste estudo de coorte (Figura Central).

### Classificação da pressão arterial

Os entrevistadores do CHARLS foram até a casa de cada participante e mediram a PA. Após o participante ter descansado por pelo menos 10 minutos, a PAS e a PAD foram medidas no braço esquerdo do participante de acordo com procedimentos padrão, e três vezes com pelo menos 45 segundos de intervalo no dia da entrevista do estudo.^14,15^ De acordo com a diretriz ACC/AHA de 2017,^8^ todos os indivíduos foram divididos em quatro grupos: grupo PA normal (PAS<120 mmHg e PAD<80 mmHg), grupo PA elevada (PAS: 120‐129 mmHg e PAD<80 mmHg), grupo hipertensão estágio 1 ( PAS: 130-139 mm Hg ou PAD: 80-89 mm Hg) e grupo de hipertensão estágio 2 (PAS≥ 140 mm Hg ou PAD ≥90 mm Hg).

## Resultados

O resultado deste estudo foi o risco de DCV e o risco de DCVA em 10 anos evento. Semelhante a estudos anteriores, a DCV foi avaliada pelas seguintes perguntas: Um médico lhe disse que você foi diagnosticado com um acidente vascular cerebral” ou “Um médico lhe disse que você foi diagnosticado com um ataque cardíaco, angina, doença coronariana, insuficiência cardíaca ou outros problemas cardíacos?” Os participantes que responderam “sim” à pergunta durante o período de acompanhamento foram definidos como portadores de DCV.^16^

De acordo com as diretrizes ACC/AHA de 2019,O risco de DCVA (%) foi calculado com base na idade, sexo, etnia, colesterol total (CT), colesterol de lipoproteína de alta densidade (HDL-C), PAS, diabetes, tratamento para hipertensão e tabagismo.^17^ Um estimador de risco DCVA Plus online: https://tools.acc.org/dcva-risk-estimator-plus/#!/calculate/estimate/. Todos os pacientes foram classificados como de baixo risco (DCVA<7,5%) e alto risco (DCVA≥ 7,5%) neste estudo.

### Covariáveis potenciais

Extraímos características gerais dos participantes: idade (anos), sexo, altura (cm), peso (kg), consumo de álcool, tabagismo, comorbidades (diabetes, depressão e dislipidemia), histórico de medicação (anti-hipertensivos, hipolipemiantes, insulina e hipoglicemiantes) e indicadores laboratoriais [CT (mg /dL), triglicerídeos (TG, mg/dL), lipoproteína de baixa densidade colesterol (LDL-C, mg/dL), HDL-C (mg/dL) e glicose (mg/dL)]. O índice de massa corporal (IMC, kg/m^2^) foi calculado pelo peso em quilogramas dividido pela altura em metros ao quadrado. Diabetes foi definido da seguinte forma: glicose plasmática em jejum (FPG) ≥7,0 mmol/L (126 mg/dL), glicose plasmática aleatória ≥11,1 nm/L (200 mg/dL), hemoglobina glicosilada (HbA1c) ≥48 nm/mol, história autorreferida de diabetes ou uso de medicação antidiabética.

### Análise estatística

Análise descritiva dos dados: empregou-se o Teste Shapiro-Wilk para avaliar a normalidade das variáveis contínuas, onde nível de significância abaixo de 0,05 indica distribuição assimétrica. Neste estudo, todas as variáveis contínuas exibiram distribuições assimétricas (Tabela 1). Para representar a distribuição dos dados contínuos, utilizamos a mediana e o intervalo interquartil [M (Q1, Q3)], enquanto as comparações entre os grupos foram realizadas usando o teste de Kruskal-Wallis semempregando testes post hoc. As variáveis categóricas foram representadas pelo número de casos e razão de composição n (%), e o teste χ^2^ foi aplicado para comparação entre grupos.

**Tabela 1:** – Características basais de todos os participantes

Variáveis	Total (n=1945)	Grupo PA normal (n=677)	Grupo PA elevada (n=269)	Grupo de hipertensão estágio 1 (n=585)	Grupo de hipertensão estágio 2 (n=414)	p	Teste de normalidade
Idade, anos, M (Q1, Q3)	56,00 (51,00,62,00)	54,00 (50,00,60,00)	56,00 (51,00,62,00)	56,00 (51,00,62,00)	58,00 (52,00,66,00)	<0,001	<0,0001
Idade, anos, n (%)						<0,001	
<60	1286 (66,12)	500 (73,86)	176 (65,43)	377 (64,44)	233 (56,28)		
≥60	659 (33,88)	177 (26.14)	93 (34,57)	208 (35,56)	181 (43,72)		
Gênero, n (%)						0,786	
Masculino	913 (46,94)	317 (46,82)	122 (45,35)	284 (48,55)	190 (45,89)		
Feminino	1032 (53.06)	360 (53,18)	147 (54,65)	301 (51,45)	224 (54,11)		
Altura, cm, M (Q1, Q3)	156,40 (151,00.163,10)	157,00 (151,20.163,00)	157,10 (150,30.163,00)	156,70 (151,60.164,60)	155,25 (150,00.162,00)	0,382	0,0001
Peso, kg, M (Q1, Q3)	56,00 (49,50,63,30)	54,00 (48,10,60,50)	55,60 (49,60,62,90)	58,10 (51,00,66,20)	57,20 (49,50,64,70)	0,061	<0,0001
IMC, kg/m^2^, M (Q1, Q3)	22,66 (20,52,25,21)	21,78 (19,91,23,97)	22,41 (20,69,25,23)	23,25 (21,17,26,14)	23,43 (21,05,26,25)	0,448	<0,0001
Bebida, n (%)	652 (33,52)	239 (35,30)	80 (29,74)	204 (34,87)	129 (31.16)	0,240	
Tabagismo, n (%)	1335 (68,64)	465 (68,69)	197 (73,23)	388 (66,32)	285 (68,84)	0,251	
Diabetes, n (%)	44 (2,26)	10 (1,48)	5 (1,86)	16 (2,74)	13 (3.14)	0,249	
Dislipidemia, n (%)	74 (3,80)	19 (2,81)	10 (3,72)	26 (4,44)	19 (4,59)	0,362	
Depressão, n (%)	13 (0,67)	4 (0,59)	3 (1,12)	2 (0,34)	4 (0,97)	0,428	
Medicamentos anti-hipertensivos, n (%)	296 (15,22)	34 (5.02)	23 (8,55)	120 (20,51)	119 (28,74)	<0,001	
Insulina e medicamentos hipoglicemiantes, n (%)	44 (2,26)	10 (1,48)	5 (1,86)	16 (2,74)	13 (3.14)	0,249	
Medicamentos hipolipemiantes, n (%)	71 (3,65)	19 (2,81)	9 (3,35)	24 (4.10)	19 (4,59)	0,420	
CT, mg/dL, M (Q1, Q3)	190,98 (167,40.214,95)	187,89 (166,62.208,76)	190,21 (161,60.212,24)	193,69 (168,17.219,98)	195,62 (171,65.220,36)	0,421	<0,0001
TG, mg/dL, M (Q1, Q3)	100,89 (73,46.146,02)	92,93 (69,03.129,21)	97,35 (70,80.144,26)	107,08 (77,88.150,45)	107,97 (77,00.163,73)	0,137	<0,0001
LDL, mg/dL, M (Q1, Q3)	114,05 (93,94.136,47)	111,73 (93,17.132,22)	111,73 (90,46.130,67)	115,98 (94,72.139,56)	117,53 (97,04.141,50)	0,635	<0,0001
HDL, mg/dL, M (Q1, Q3)	50,64 (40,98,60,31)	52,58 (42,91,61,47)	51,03 (40,98,61,08)	49,10 (40,59,59,15)	48,33 (40,21,59,92)	0,209	<0,0001
Glicose, mg/dL, M (Q1, Q3)	100,98 (93,06.111,60)	98,46 (91,80.107,82)	100,80 (92,16.111,42)	101,88 (94,32.112,86)	105,12 (94,68.116,28)	0,062	<0,0001

*IMC: índice de massa corporal; CT: colesterol total; TG: triglicerídeos; LDL: lipoproteína de baixa densidade; HDL: lipoproteína de alta densidade*

A regressão univariada de COX foi adotada para rastrear alguns fatores de confusão relacionados à DCV. Para examinar a relação da classificação de PA ACC/AHA de 2017 com o risco de DCV em pessoas de meia-idade e idosos na China, realizamos modelos de regressão COX univariados e multivariados. Modelo 1: modelo de regressão COX univariado (sem ajuste); Modelo 2: idade, sexo e IMC ajustados; Modelo 3: idade ajustada, sexo, escolaridade, consumo de álcool, IMC, diabetes, anti-hipertensivos, TG, LDL-C, HDL-C e glicose. Foi calculada uma taxa de risco (HR) com IC95%. Utilizou a análise de regressão logística univariada para rastrear alguns fatores de confusão relacionados ao risco de DCVA em 10 anos. Realizamos modelos de regressão logística univariada e multivariada para avaliar a relação da classificação PA ACC/AHA de 2017 com o risco de DCV e o risco de DCVA em 10 anos. Modelo 4: modelo de regressão logística univariada (sem ajuste); Modelo 5: idade, sexo e IMC ajustados; Modelo 6: idade ajustada, sexo, escolaridade, consumo de álcool, tabagismo, IMC, diabetes, anti-hipertensivos, TG, LDL-C, HDL-C e glicose. Posteriormente, também realizamos análises de subgrupos com base na idade, sexo e uso de medicamentos anti-hipertensivos. O odds ratio (OR) e o intervalo de confiança (IC) de 95% foram calculados neste estudo. Todas as análises foram realizadas utilizando os softwares estatísticos RStudio 4.0.3 e SAS 9.4, e p<0,05 foi aceito como estatisticamente significativo.

## Resultados

### Características base

**Figura 1:**
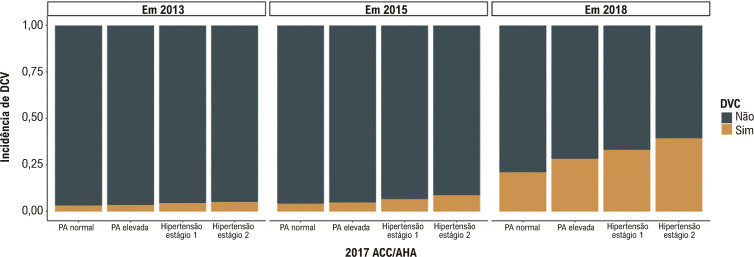
– A incidência de DCV em 2, 4 e 7 anos em diferentes grupos de pressão arterial. DCV: doença cardiovascular.

**Figura 2:**
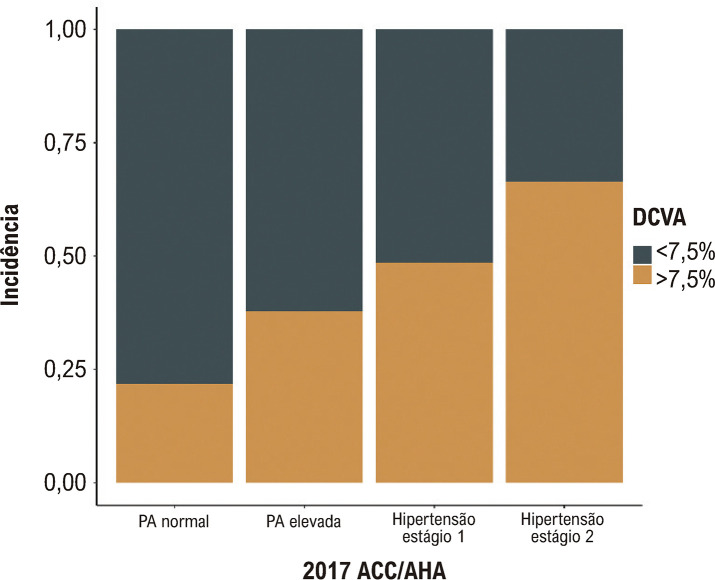
– A incidência de DCVA em diferentes grupos de pressão arterial. DCVA: doença cardiovascular aterosclerótica.

Para avaliar o impacto dos níveis de PA no risco de DCV, excluímos adicionalmente os participantes que perderam o acompanhamento antes de 2018 (n=4.699). A Tabela 1 mostra as características iniciais de 1.945 participantes. Todos os indivíduos foram divididos em quatro grupos seguindo a diretriz ACC/AHA de 2017: grupo de PA normal, grupo de PA elevada, grupo de hipertensão estágio 1 e grupo de hipertensão estágio 2. Obviamente, em comparação com outros grupos, incluindo PA normal, PA elevada e hipertensão estágio 1, os indivíduos com hipertensão estágio 2 pareciam ser mais velhos e tinham um IMC mais elevado. A Figura 1 também mostra que à medida que a PA aumenta, também aumenta a incidência de DCV em 2013, 2015 e 2018. Além disso, para aqueles com maior risco de DCVA (DCVA≥7,5%), encontramos uma correlação positiva entre PA elevada e um risco aumentado de DCVA em 10 anos (Figura 2).

### Relação entre a classificação de PA ACC/AHA de 2017 e o risco de DCV/DCVA

A Tabela 2 mostra a relação entre a classificação de PA da ACC/AHA de 2017 com o risco de DCV dos pacientes e risco aumentado de DCVA em 10 anos. Após ajustar todas as covariáveis (Modelo 2), em comparação com pacientes de meia-idade e idosos com PA normal, descobrimos que pacientes com hipertensão estágio 1 e hipertensão estágio 2 estavam associados a um maior risco de DCV, separadamente.

**Tabela 2:** – Relação entre a classificação da PA 2017ACC/AHA e o risco de DCV/DCVA

Resultados 1	Variáveis	Tamanho da amostra, n (%)	Modelo 1		Modelo 2		Modelo 3	
			HR (IC 95%)	p	HR (IC 95%)	p	HR (IC 95%)	p
DCV	PA normal	147 (21,71)	Referência		Referência		Referência	
	PA elevada	78 (29,00)	1.382 (1.050-1.819)	0,021	1,279 (0,971-1,686)	0,080	1,246 (0,944-1,643)	0,120
	Hipertensão estágio 1	201 (34,36)	1.702 (1.376-2.105)	<0,001	1.526 (1.228-1.896)	<0,001	1.379 (1.105-1.720)	0,004
	Hipertensão estágio 2	166 (40,10)	2.076 (1.662-2.592)	<0,001	1.735 (1.381-2.179)	<0,001	1.479 (1.168-1.874)	0,001
**Resultados 2**	**Variáveis**		**Modelo 4**		**Modelo 5**		**Modelo 6**	
			**OR (IC95%)**	**p**	**OR (IC95%)**	**p**	**OR (IC95%)**	**p**
DCVA (≥7,5%)	PA normal	/	Referência		Referência		Referência	
	PA elevada	/	2.178 (1.848-2.568)	<0,001	1.826 (1.213-2.749)	0,004	2.048 (1.645-2.550)	<0,001
	Hipertensão estágio 1	/	3.371 (2.951-3.851)	<0,001	4.540 (3.190-6.459)	<0,001	3.627 (3.023-4.352)	<0,001
	Hipertensão estágio 2	/	7.079 (6.096-8.221)	<0,001	13.881 (9.003-21.401)	<0,001	7.920 (6.410-9.786)	<0,001

*PA: pressão arterial; HR: razão de risco; OR: razão de chances; IC: intervalo de confiança; ACC/AHA: American College of Cardiology/American Heart Association; DCVA:doença cardiovascular aterosclerótica; DCV: doença cardiovascular. Modelo 1: modelo de regressão COX univariado (sem ajuste); Modelo 2: idade ajustada, sexo, índice de massa corporal. Modelo 3: idade ajustada, sexo, escolaridade, consumo de álcool, índice de massa corporal, diabetes, anti-hipertensivos, triglicerídeos, colesterol de lipoproteína de baixa densidade, colesterol de lipoproteína de alta densidade e glicose. Modelo 4: modelo de regressão logística univariada (sem ajuste); Modelo 5: idade ajustada, sexo, índice de massa corporal; Modelo 6: idade ajustada, sexo, escolaridade, consumo de álcool, tabagismo, índice de massa corporal, diabetes, anti-hipertensivos, triglicerídeos, colesterol de lipoproteína de baixa densidade, colesterol de lipoproteína de alta densidade e glicose.*

Simultaneamente, também observamos uma associação positiva entre indivíduos com PA elevada, hipertensão estágio 1, hipertensão estágio 2 e maior risco de DCVA no modelo totalmente ajustado (Tabela 2).

### Análises de subgrupos baseadas em idade, sexo e uso de medicamentos anti-hipertensivos

Analisamos a relação entre os níveis de PA e o risco de DCV/DCVA em diferentes populações. Conforme mostrado na Tabela 3, para pessoas de meia-idade e idosos de diferentes idades, a hipertensão estágio 1/2 foi associada a um risco aumentado de DCV (p<0,05). A relação entre hipertensão estágio 1/2 e DCV permaneceu nos subgrupos femininos. Entre a população masculina, a hipertensão em estágio 2 foi associada a um risco aumentado de DCV com significância estatística (p=0,023), e a associação entre hipertensão em estágio 1 e risco de DCV tem significância marginal (p=0,057). Entre participantes de meia-idade e idosos sem uso de medicamentos anti-hipertensivos, tanto a hipertensão estágio 1 quanto a hipertensão estágio 2 foram relacionadas a um risco aumentado de DCV. Entretanto, a relação entre os níveis de PA e DCV não foi estatisticamente significativa em pessoas de meia-idade e idosos em uso de anti-hipertensivos. Notavelmente, uma relação positiva entre diferentes níveis de PA e maior risco de DCVA foi observada em todas as análises de subgrupos.

**Tabela 3:** – Análises de subgrupos com base em idade, sexo e uso de anti-hipertensivos

Análises de subgrupos	Variáveis	DCV de 7 anos		DCVA(≥7,5%)	
		HR (IC 95%)	p	OR (IC95%)	p
Análise de subgrupo I: Idade<60	PA normal	Referência		Referência	
	PA elevada	1,176 (0,820-1,686)	0,377	1,539 (0,888-2,669)	0,124
	Hipertensão estágio 1	1.388 (1.048-1.837)	0,022	3.200 (2.048-4.999)	<0,001
	Hipertensão estágio 2	1.886 (1.403-2.535)	<0,001	12.486 (6.905-22.576)	<0,001
Análise de subgrupo I: Idade≥60	PA normal	Referência		Referência	
	PA elevada	1,384 (0,892-2,148)	0,147	3.806 (1.608-9.007)	0,002
	Hipertensão estágio 1	1.593 (1.107-2.292)	0,012	5.261 (2.446-11.318)	<0,001
	Hipertensão estágio 2	1.510 (1.039-2.194)	0,031	19.245 (7.168-51.668)	<0,001
Análise de subgrupo II: Masculino	PA normal	Referência	-	Referência	
	PA elevada	1,178 (0,820-1,692)	0,375	3.449 (1.878-6.336)	<0,001
	Hipertensão estágio 1	1,325 (0,991-1,772)	0,057	14.612 (8.635-24.727)	<0,001
	Hipertensão estágio 2	1.429 (1.049-1.945)	0,023	67.329 (37.550-120.726)	<0,001
Análise de subgrupo II: Feminino	PA normal	Referência	-	Referência	
	PA elevada	1,392 (0,903-2,148)	0,135	3.246 (2.380-4.426)	<0,001
	Hipertensão estágio 1	1.591 (1.130-2.239)	0,008	4.902 (3.760-6.392)	<0,001
	Hipertensão estágio 2	1.931 (1.350-2.762)	<0,001	12.288 (8.695-17.365)	<0,001
Análise de subgrupo III: Sem uso de medicamentos anti-hipertensivos	PA normal	Referência	-	Referência	
	PA elevada	1,287 (0,960-1,726)	0,091	2.963 (2.229-3.938)	<0,001
	Hipertensão estágio 1	1.359 (1.064-1.736)	0,014	6.110 (4.777-7.815)	<0,001
	Hipertensão estágio 2	1.504 (1.149-1.967)	0,003	23.147 (16.768-31.952)	<0,001
Análise de subgrupo III: Uso de medicamentos anti-hipertensivos	PA normal	Referência	-	Referência	
	PA elevada	0,846 (0,353-2,029)	0,708	3.229 (1.264-8.247)	0,014
	Hipertensão estágio 1	1,265 (0,698-2,295)	0,439	4.631 (2.178-9.847)	<0,001
	Hipertensão estágio 2	1,260 (0,696-2,281)	0,445	12.992 (5.921-28.505)	<0,001

*PA: pressão arterial; HR: razão de risco; OR: razão de chances; IC: intervalo de confiança; ACC/AHA: American College of Cardiology/American Heart Association; DCVA: doença cardiovascular aterosclerótica; DCV: doença cardiovascular. Para DCV: idade ajustada (não ajustada na análise de subgrupo I), gênero (não ajustado na análise de subgrupo II), escolaridade, consumo de álcool, índice de massa corporal, diabetes, medicamentos anti-hipertensivos (não ajustado na análise de subgrupos III), triglicerídeos, colesterol de lipoproteína de baixa densidade, colesterol de lipoproteína de alta densidade e glicose. Para DCVA: idade ajustada (não ajustada na análise de subgrupo I), gênero (não ajustado na análise de subgrupo II), escolaridade, consumo de álcool, tabagismo, índice de massa corporal, diabetes, medicamentos anti-hipertensivos (não ajustado na análise de subgrupos III), triglicerídeos, colesterol de lipoproteína de baixa densidade, colesterol.*

## Discussão

Este estudo de coorte utilizou os dados do banco CHARLS para observar que pacientes com hipertensão em estágio 1/2 definida pela diretriz de hipertensão ACC/AHA de 2017 estavam associados a um maior risco de DCV em comparação com a PA normal entre pessoas de meia-idade e idosos na China. Além disso, houve uma associação positiva entre indivíduos com PA elevada, hipertensão estágio 1, hipertensão estágio 2 e risco de DCVA em 10 anos.

Em novembro de 2017, a ACC e a AHA divulgaram uma diretriz clínica para a prevenção, detecção e tratamento da hipertensão.^18^ Ao contrário do Sétimo Relatório do *Joint National Committee* (JNC7) de 2003, que definiu hipertensão como PAS≥140 mmHg ou PAD≥90 mmHg na população em geral,^19^ as diretrizes da ACC/AHA de 2017 recomendam o uso de um limiar de PA mais baixo para diagnosticar hipertensão.^20^ Evidências recentes sugerem que a diretriz de hipertensão ACC/AHA de 2017 aumentou substancialmente a prevalência de hipertensão.^21,22^ A aplicação das diretrizes da ACC/AHA de 2017 tem sido um tema de preocupação global, particularmente o impacto dos níveis de PA no risco de DCV. Até agora, vários estudos foram realizados para avaliar a associação entre os níveis de PA definidos pela diretriz de hipertensão ACC/AHA de 2017 e a incidência de DCV.^9-11^ No entanto, esses resultados têm sido inconsistentes devido à seleção da população. Um estudo retrospectivo incluindo 15.508.537 participantes coreanos com idades entre 20 e 39 anos demonstrou que a hipertensão em estágio 1 está associada a um risco maior de DCV.^23^ Um estudo de coorte multiprovincial na China mostrou que a hipertensão em estágio 1 definida pela diretriz de hipertensão ACC/AHA de 2017 não estava relacionada ao risco de DCV em participantes com idade ≥60 anos.^10^ Da mesma forma, um estudo de coorte do norte da China ilustrou que a hipertensão em estágio 1 [taxa de risco = 1,25, IC 95%: 1,11-1,40] apresentava um risco maior de eventos cardiovasculares em comparação com a PA normal.^24^ Em comparação, nosso estudo é o primeiro realizado na China a investigar a relação entre os níveis de PA e o risco de DCV/DCVA em pessoas de meia-idade e idosos (≥45 anos) com base no banco de dados CHARLS.

No presente estudo, após ajuste dos fatores de confusão, a hipertensão estágio 1/2 foi correlacionada com um risco aumentado de DCV em 7 anos. Esses resultados indicaram que PAS ≥130 mmHg ou PAD ≥80 mmHg foi considerada um fator de risco para DCV de longo prazo em pessoas de meia-idade e idosas. No entanto, de acordo com as diretrizes de PA do CHL de 2018, PAS≥140 mmHg e/ou PAD≥90 mmHg é comumente usada para definir hipertensão na população chinesa. A hipertensão tem sido reconhecida como um fator de risco para DCV. Em outras palavras, a classificação da PA de acordo com as diretrizes de PA da ACC/AHA de 2017 pode ser aplicada à população chinesa. Mais pesquisas são necessárias para verificar as descobertas no futuro. Pesquisas anteriores demonstraram que a avaliação do risco de DCVA é um passo crucial no manejo da prevenção de DCV. Este estudo sugere uma correlação positiva entre PA elevada e risco aumentado de DCVA elevada em 10 anos. Portanto, é importante permanecer vigilante para a ocorrência de DCVA de alto risco em pacientes com PA mais elevada (PAS> 120 mm Hg ou PAD> 80 mm Hg).

Um estudo de coorte prospectivo da China mostrou que a hipertensão em estágio 1 estava associada a um risco aumentado de acidente vascular cerebral em mulheres rurais com idade ≥ 45 anos, e a hipertensão em estágio 2 estava associada a um risco significativamente aumentado de acidente vascular cerebral em mulheres com mais de 35 anos de idade em comparação com a PA normal.^25^ Neste estudo, descobrimos que a hipertensão em estágio 1/2 exerceu um impacto significativo no risco de DCV a longo prazo em homens e mulheres chineses.

Nosso estudo tem vários pontos fortes. Usamos o banco de dados CHARLS que era uma amostra representativa nacionalmente de adultos chineses de meia e mais idade, e os resultados podem ter ampla generalização na China. Além disso, a PA foi medida de forma objetiva e não autorreferida no presente estudo. No entanto, este estudo também tem várias limitações. Em primeiro lugar, uma vez que todos os dados deste estudo foram derivados da base de dados CHARLS, o diagnóstico de DCV e diabetes baseou-se nos auto-relatos dos participantes, o que pode subestimar a incidência real de DCV e diabetes. Em segundo lugar, embora tenhamos ajustado várias covariáveis que podem confundir a relação entre o risco de DCV e os níveis de PA entre pessoas de meia-idade e idosos, alguns fatores de confusão, como indicadores laboratoriais, hábitos de vida, atividade física e histórico familiar de hipertensão não foram captados em este estudo. Mais estudos prospectivos precisam ser realizados no futuro para explorar esta associação entre o risco de DCV e os níveis de PA definidos pela diretriz de hipertensão ACC/AHA de 2017. Em terceiro lugar, o resultado deste estudo baseou-se numa população de adultos chineses de meia-idade e mais velhos, pelo que os nossos resultados podem não se aplicar a populações de outros países.

## Conclusão

Em conclusão, este estudo indicou que a classificação da PA de acordo com as diretrizes de PA da ACC/AHA de 2017 pode ser aplicada à população chinesa. Quando PAS ≥130 mmHg ou PAD ≥80 mmHg, pessoas de meia-idade e idosos podem ter maior risco de DCV. Além disso, deve ser dada maior atenção a indivíduos de meia-idade e idosos com PA elevada (PAS >120 mmHg ou PAD >80 mmHg) devido ao seu potencial elevado risco de DCVA.
