# Supplementary material for: A Associação de Pressão Arterial Definida pelas Diretrizes ACC/AHA de 2017 e Risco de Doença Cardiovascular para Pessoas de Meia-Idade e Idosas na China: Um Estudo de Coorte
Source: Arq Bras Cardiol. 2024 Aug 6;121(7):e20230785. [Article in Portuguese] doi: 10.36660/abc.20230785 (PMC11341210; doi:10.36660/abc.20230785)
Supplement: Translation

# Translation: The Association of Blood Pressure Defined by the 2017 ACC/AHA Guidelines and Cardiovascular Disease Risk for Middle-Aged and Elderly People in China: A Cohort Study

**DOI:** 10.36660/abc.20230785i

**Authors:** Qingyang Lu, Haijing Xie, Xuefeng Gao

**Affiliations:** 1 China Xicheng District Guangwai Hospital - Cardiovascular Medicine, Beijing – China; 2 China Chongming Hospital Affiliated to Shanghai University of Medicine and Health Sciences - Oncology, Shanghai – China; 3 China Academician of the World Academy of Productivity Sciences, Changchun – China

**Keywords:** Cardiovascular Diseases, Hypertension, Middle Aged, Aged

## Abstract

**Background:**

Cardiovascular disease (CVD) is a series of diseases affecting the heart or blood vessels.

**Objectives:**

To assess the relationship between blood pressure (BP) levels defined by the 2017 American College of Cardiology/American Heart Association (ACC/AHA) guideline and CVD/atherosclerotic cardiovascular disease (ASCVD) risk for middle-aged and elderly people in China.

**Methods:**

A total of 6,644 middle-aged and elderly people from the China Health and Retirement Longitudinal Study (CHARLS) were finally included. According to the 2017 ACC/AHA guideline, all subjects were divided into four groups: normal BP, elevated BP, stage 1 hypertension, and stage 2 hypertension. The outcome of this study was considered as the risk of CVD and ASCVD. Univariate and multivariate COX regression models were adopted to examine the relationship of the 2017 ACC/AHA BP classification with the risk of CVD. Univariate and multivariate logistic regression models were used to investigate the association between BP levels and ASCVD risk. Subgroup analyses based on age, gender, and use of antihypertensive drugs were performed. P<0.05 was accepted as statistically significant.

**Results:**

After adjusting all covariates, compared to middle-aged and elderly patients with normal BP, we found that patients with stage 1/2 hypertension were associated with a higher risk of CVD, separately. Simultaneously, we also observed a positive association between individuals with elevated BP, stage 1 hypertension, stage 2 hypertension, and higher ASCVD risk in the fully adjusted model. The result of subgroup analyses implied that the relationship between stage 1/2 hypertension and CVD/ high ASCVD was robust in different ages and genders, and participants without using antihypertensive drugs.

**Conclusion:**

BP classification under the 2017 ACC/AHA BP guidelines may apply to the Chinese population.

## Introduction

Cardiovascular disease (CVD) is a series of diseases affecting the heart or blood vessels.^1^ It is currently recognized as the leading cause of death globally, and accounts for over 40% of deaths in China.^2^ In China, the prevalence of CVD is still on the rise with socioeconomic development, an aging population, and lifestyle changes.^3,4^ The prevalence of CVD is estimated to have doubled since 1990, reaching nearly 94 million in 2016, which has imposed a burden on health and economic costs.^2^ Therefore, understanding the impact of CVD risk factors is critical to optimize CVD prevention measures.

It is common knowledge that hypertension is significantly associated with the risk of CVD.^5^ Evidence shows that middle-aged and elderly people are more likely to suffer from hypertension.^6^ Effective management of hypertension plays an important role in preventing the prevalence of CVD for middle-aged and elderly people. At present, systolic blood pressure (SBP) ≥140 mmHg and/or diastolic blood pressure (DBP)≥90 mmHg is commonly used to define hypertension in the Chinese population based on the 2018 Chinese Hypertension League (CHL) blood pressure (BP) guidelines.^7^ However, in 2017, the American College of Cardiology/American Heart Association (ACC/AHA) released an updated guideline about new diagnostic criteria for hypertension:^8^ stage 1 hypertension was defined as systolic blood pressure (SBP) with 130-139 mmHg or diastolic blood pressure (DBP) with 80-89 mmHg; stage 2 hypertension was defined as SBP≥140 mmHg or DBP≥90 mmHg. ACC/AHA guidelines may overestimate the prevalence and number of patients with hypertension. Whether the 2017 ACC/AHA guidelines apply to the Chinese population remains unclear. Several studies have investigated the association between hypertension defined by the 2017 ACC/AHA guidelines and CVD risk among the Chinese population.^9-11^ An epidemiological study performed among adults aged 35-49 years in rural areas, in China showed that stage 1 hypertension defined by the 2017 ACC/AHA guidelines was associated with a higher risk of stroke.^11^ In the study of Qi Y, et al., they reported that the 2017 ACC/AHA stage 1 hypertension was related to cardiovascular risk among young and middle-aged Chinese adults (aged 35-59 years), but not in those ≥60 years of age.^9^ In addition, Xie YX, et al., also pointed out that controlling BP in elderly Chinese patients (≥60 years) with stage 1 hypertension may help to reduce the risk of CVD.^10^ Clearly, the results of studies on the relationship between hypertension under the new criteria and the risk of CVD for the Chinese population were still controversial so far.

Herein, the present study aimed to assess the relationship between the 2017 ACC/AHA BP classification and the risk of CVD for middle-aged and elderly people in China based on the China Health and Retirement Longitudinal Study (CHARLS) database. Additionally, we also further explored the relationship between the 2017 ACC/AHA BP classification and atherosclerotic cardiovascular disease (ASCVD) risk.

## Methods

### Study population

All data of this study was derived from the CHARLS database. The CHARLS adopted a multistage sampling strategy covering 28 provinces, 150 counties, and 450 villages/urban communities, which collected subjects’ information on personal information, family, health status, physical measurement, utilization of medical services and health insurance, work, retirement and pensions, income, consumption, assets, and community information.^12^ The CHARLS baseline data was collected in 2011, wave 2 in 2013, wave 3 in 2015 and wave 4 in 2018.^13^ The CHARLS was approved by the Institutional Review Committee of Peking University. All participants received written informed consent.

For this cohort study, we selected participants from the CHARLS database in 2011 (n=15,264). We excluded some individuals who met the following criteria: (1) participants with a history of CVD at baseline (n=2,237); (2) participants with missing SBP or DBP records at baseline (n=2,996); (3) participants with missing data on participants’ demographic information, comorbidity, medication history, and laboratory indicators (n=3,387). A total of 6,644 eligible adults aged ≥45 years were included in this cohort study (Central Illustration).

### Blood pressure classification

The CHARLS interviewers went to each participant’s home and measured the BP. After the participant had rested for at least 10 minutes, SBP and DBP were measured on the participant’s left arm according to standard procedures, and three times at least 45 seconds apart on the day of the study interview.^14,15^ According to the 2017 ACC/AHA guideline,^8^ all subjects were divided into four groups: normal BP group (SBP<120 mm Hg and DBP<80 mm Hg), elevated BP group (SBP: 120–129 mm Hg and DBP<80 mm Hg), stage 1 hypertension group (SBP: 130–139 mm Hg or DBP: 80–89 mm Hg), and stage 2 hypertension group (SBP≥ 140 mm Hg or DBP ≥90 mm Hg).

### Outcomes

The outcome of this study was the CVD risk and 10-year risk of ASCVD event. Similar to previous studies, CVD was assessed by the following questions: Have you been told by a doctor that you have been diagnosed with a stroke” or “Have you been told by a doctor that you have been diagnosed with a heart attack, angina, coronary heart disease, heart failure, or other heart problems?” Participants who answered “yes” to the question during the follow-up period were defined as having CVD.^16^

According to 2019 ACC/AHA guidelines, ASCVD risk (%) was computed based on age, gender, ethnicity, total cholesterol (TC), high-density lipoprotein cholesterol (HDL-C), SBP, diabetes, treatment for hypertension, and smoking status.^17^ An online ASCVD Risk Estimator Plus: https://tools.acc.org/ascvd-risk-estimator-plus/#!/calculate/estimate/. All patients were classified as low-risk (ASCVD<7.5%) and high-risk (ASCVD≥ 7.5%) in this study.

### Potential covariates

We extracted general characteristics of participants: age (years), gender, height (cm), weight (kg), drinking, smoking, comorbidity (diabetes, depression, and dyslipidemia), medication history (antihypertensive drugs, hypolipidemic drugs, insulin, and hypoglycemic drugs) and laboratory indicators [TC (mg/dL), triglycerides (TG, mg/dL), low-density lipoprotein cholesterol (LDL-C, mg/dL), HDL-C (mg/dL) and glucose (mg/dL)]. Body mass index (BMI, kg/m^2^) was calculated by weight in kilograms divided by height in meters squared. Diabetes was defined as follows: fasting plasma glucose (FPG) ≥7.0 mmol/L (126 mg/dL), random plasma glucose ≥11.1 nm/L (200 mg/dL), glycosylated hemoglobin (HbA1c) ≥48 nm/mol, self-reported history of diabetes, or the use of anti-diabetic medication.

### Statistical analysis

Descriptive analysis of data: we employed the Shapiro-Wilk Test to assess the normality of continuous variables, where a significance level below 0.05 indicates a skewed distribution. In this study, all continuous variables exhibited skewed distributions (Table 1). To depict the distribution of continuous data, we utilized the median and interquartile range [M (Q1, Q3)], while group comparisons were conducted using the Kruskal-Wallis test without employing post hoc tests. Categorical variables were depicted by the number of cases and composition ratio n (%), and the χ^2^ test was applied for the comparison between groups.

**Table 1:** – The baseline characteristics of all participants

Variables	Total (n=1945)	Normal BP group (n=677)	Elevated BP group (n=269)	Stage 1 hypertension group (n=585)	Stage 2 hypertension group (n=414)	p	Normality test
Age, years, M (Q1, Q3)	56.00 (51.00,62.00)	54.00 (50.00,60.00)	56.00 (51.00,62.00)	56.00 (51.00,62.00)	58.00 (52.00,66.00)	<0.001	<0.0001
Age, years, n (%)						<0.001	
<60	1286 (66.12)	500 (73.86)	176 (65.43)	377 (64.44)	233 (56.28)		
≥60	659 (33.88)	177 (26.14)	93 (34.57)	208 (35.56)	181 (43.72)		
Gender, n (%)						0.786	
Male	913 (46.94)	317 (46.82)	122 (45.35)	284 (48.55)	190 (45.89)		
Female	1032 (53.06)	360 (53.18)	147 (54.65)	301 (51.45)	224 (54.11)		
Height, cm, M (Q1, Q3)	156.40 (151.00,163.10)	157.00 (151.20,163.00)	157.10 (150.30,163.00)	156.70 (151.60,164.60)	155.25 (150.00,162.00)	0.382	0.0001
Weight, kg, M (Q1, Q3)	56.00 (49.50,63.30)	54.00 (48.10,60.50)	55.60 (49.60,62.90)	58.10 (51.00,66.20)	57.20 (49.50,64.70)	0.061	<0.0001
BMI, kg/m^2^, M (Q1, Q3)	22.66 (20.52,25.21)	21.78 (19.91,23.97)	22.41 (20.69,25.23)	23.25 (21.17,26.14)	23.43 (21.05,26.25)	0.448	<0.0001
Drinking, n (%)	652 (33.52)	239 (35.30)	80 (29.74)	204 (34.87)	129 (31.16)	0.240	
Smoking, n (%)	1335 (68.64)	465 (68.69)	197 (73.23)	388 (66.32)	285 (68.84)	0.251	
Diabetes, n (%)	44 (2.26)	10 (1.48)	5 (1.86)	16 (2.74)	13 (3.14)	0.249	
Dyslipidemia, n (%)	74 (3.80)	19 (2.81)	10 (3.72)	26 (4.44)	19 (4.59)	0.362	
Depression, n (%)	13 (0.67)	4 (0.59)	3 (1.12)	2 (0.34)	4 (0.97)	0.428	
Antihypertensive drugs, n (%)	296 (15.22)	34 (5.02)	23 (8.55)	120 (20.51)	119 (28.74)	<0.001	
Insulin and hypoglycemic drugs, n (%)	44 (2.26)	10 (1.48)	5 (1.86)	16 (2.74)	13 (3.14)	0.249	
Hypolipidemic drugs, n (%)	71 (3.65)	19 (2.81)	9 (3.35)	24 (4.10)	19 (4.59)	0.420	
TC, mg/dL, M (Q1, Q3)	190.98 (167.40,214.95)	187.89 (166.62,208.76)	190.21 (161.60,212.24)	193.69 (168.17,219.98)	195.62 (171.65,220.36)	0.421	<0.0001
TG, mg/dL, M (Q1, Q3)	100.89 (73.46,146.02)	92.93 (69.03,129.21)	97.35 (70.80,144.26)	107.08 (77.88,150.45)	107.97 (77.00,163.73)	0.137	<0.0001
LDL, mg/dL, M (Q1, Q3)	114.05 (93.94,136.47)	111.73 (93.17,132.22)	111.73 (90.46,130.67)	115.98 (94.72,139.56)	117.53 (97.04,141.50)	0.635	<0.0001
HDL, mg/dL, M (Q1, Q3)	50.64 (40.98,60.31)	52.58 (42.91,61.47)	51.03 (40.98,61.08)	49.10 (40.59,59.15)	48.33 (40.21,59.92)	0.209	<0.0001
Glucose, mg/dL, M (Q1, Q3)	100.98 (93.06,111.60)	98.46 (91.80,107.82)	100.80 (92.16,111.42)	101.88 (94.32,112.86)	105.12 (94.68,116.28)	0.062	<0.0001

*BMI: body mass index; Tc: total cholesterol; TG: triglycerides; LDL: low-density lipoprotein; HDL: high-density lipoprotein.*

Univariate COX regression was adopted to screen some confounding factors related to CVD. To examine the relationship of the 2017 ACC/AHA BP classification with the risk of CVD for middle-aged and elderly people in China, we performed univariate and multivariate COX regression models. Model 1: univariate COX regression model (no adjustment); Model 2: adjusted age, gender, and BMI; Model 3: adjusted age, gender, educational level, drinking, BMI, diabetes, antihypertensive drugs, TG, LDL-C, HDL-C, and glucose. A hazard ratio (HR) with 95%CI was calculated. Used the univariate logistic regression analysis to screen some confounding factors related to the 10-year risk of ASCVD. We performed univariate and multivariate logistic regression models to assess the relationship of the 2017 ACC/AHA BP classification with the risk of CVD and the 10-year risk of ASCVD. Model 4: univariate logistic regression model (no adjustment); Model 5: adjusted age, gender, and BMI; Model 6: adjusted age, gender, educational level, drinking, smoking, BMI, diabetes, antihypertensive drugs, TG, LDL-C, HDL-C, and glucose. Subsequently, we also conducted subgroup analyses based on the age, gender, and use of antihypertensive drugs. The odds ratio (OR) and 95% confidence interval (CI) were calculated in this study. All analyses were performed using RStudio 4.0.3 and SAS 9.4 statistical software, and *P* <0.05 was accepted as statistically significant.

## Results

### Baseline characteristics

To evaluate the impact of BP levels on CVD risk, we additionally excluded participants who were lost to follow-up before 2018 (n=4699). Table 1 shows the baseline characteristics of 1,945 participants. All subjects were divided into four groups following the 2017 ACC/AHA guideline: normal BP group, elevated BP group, stage 1 hypertension group, and stage 2 hypertension group. Obviously, compared to other groups including normal BP, elevated BP, and stage 1 hypertension, individuals with stage 2 hypertension appeared to be older, and had a higher BMI. Figure 1 also displays that as BP increases, so does the incidence of CVD in 2013, 2015, and 2018. In addition, for those with higher ASCVD risk (ASCVD≥7.5%), we found a positive correlation between elevated BP and an increased 10-year higher risk of ASCVD (Figure 2).

**Figure 1:** – The incidence of 2-year, 4-year, and 7-year CVD in different blood pressure groups. CVD: cardiovascular disease.

**Figure 2:** – The incidence of ASCVD in different blood pressure groups. ASCVD: atherosclerotic cardiovascular disease.

### Relationship of 2017 ACC/AHA BP classification and CVD/ASCVD risk

Table 2 shows the relationship between the 2017 ACC/AHA BP classification with patients’ risk of CVD and 10-year higher risk of ASCVD. After adjusting all covariates (Model 2), compared to middle-aged and elderly patients with normal BP, we found that patients with stage 1 hypertension and stage 2 hypertension were associated with a higher risk of CVD, separately.

**Table 2:** – The relationship between 2017ACC/AHA BP classification and the risk of CVD/ ASCVD

**Outcomes 1**	**Variables**	**Sample size, n (%)**	**Model 1**		**Model 2**		**Model 3**	
			**HR (95%CI)**	**p**	**HR (95%CI)**	**p**	**HR (95%CI)**	**p**
CVD	Normal BP	147 (21.71)	Ref		Ref		Ref	
	Elevated BP	78 (29.00)	1.382 (1.050-1.819)	0.021	1.279 (0.971-1.686)	0.080	1.246 (0.944-1.643)	0.120
	Stage 1 hypertension	201 (34.36)	1.702 (1.376-2.105)	<0.001	1.526 (1.228-1.896)	<0.001	1.379 (1.105-1.720)	0.004
	Stage 2 hypertension	166 (40.10)	2.076 (1.662-2.592)	<0.001	1.735 (1.381-2.179)	<0.001	1.479 (1.168-1.874)	0.001
**Outcomes 2**	**Variables**		**Model 4**		**Model 5**		**Model 6**	
			**OR (95%CI)**	**p**	**OR (95%CI)**	**p**	**OR (95%CI)**	**p**
ASCVD (≥7.5%)	Normal BP	/	Ref		Ref		Ref	
	Elevated BP	/	2.178 (1.848-2.568)	<0.001	1.826 (1.213-2.749)	0.004	2.048 (1.645-2.550)	<0.001
	Stage 1 hypertension	/	3.371 (2.951-3.851)	<0.001	4.540 (3.190-6.459)	<0.001	3.627 (3.023-4.352)	<0.001
	Stage 2 hypertension	/	7.079 (6.096-8.221)	<0.001	13.881 (9.003-21.401)	<0.001	7.920 (6.410-9.786)	<0.001

*BP: blood pressure; HR: hazard ratio; OR: odds ratio; C: confidence interval; ACC/AHA: American College of Cardiology/American Heart Association; ASCVD: atherosclerotic cardiovascular disease; CVD: cardiovascular disease. Model 1: univariate COX regression model (no adjustment); Model 2: adjusted age, gender, body mass index. Model 3: adjusted age, gender, educational level, drinking, body mass index, diabetes, antihypertensive drugs, triglycerides, low-density lipoprotein cholesterol, high-density lipoprotein cholesterol, and glucose. Model 4: univariate logistic regression model (no adjustment); Model 5: adjusted age, gender, body mass index; Model 6: adjusted age, gender, educational level, drinking, smoking, body mass index, diabetes, antihypertensive drugs, triglycerides, low-density lipoprotein cholesterol, high-density lipoprotein cholesterol, and glucose.*

Simultaneously, we also observed a positive association between individuals with elevated BP, stage 1 hypertension, stage 2 hypertension, and higher ASCVD risk in the fully adjusted model (Table 2).

### Subgroup analyses based on age, gender, and use of antihypertensive drugs

We analyzed the relationship between BP levels and the risk of CVD/ASCVD in different populations. As shown in Table 3, for middle-aged and elderly people of different ages, stage 1/2 hypertension was associated with an increased risk of CVD (p<0.05). The relationship between stage 1/ 2 hypertension and CVD remained in the female subgroups. Among the male population, stage 2 hypertension was linked with an increased risk of CVD with a statistical significance (p=0.023), and the association of stage 1 hypertension and CVD risk has a marginal significance (p=0.057). Among middle-aged and elderly participants without using antihypertensive drugs, both stage 1 and stage 2 hypertension were related to an increased risk of CVD. However, the relationship between BP levels and CVD was not statistically significant in middle-aged and elderly people with using antihypertensive drugs. Notably, a positive relationship between different BP levels and higher ASCVD risk was observed in all subgroup analyses.

**Table 3:** – Subgroup analyses based on age, gender, and use of antihypertensive drugs

Subgroup analyses	Variables	7-year CVD		ASCVD (≥7.5%)	
		HR (95%CI)	p	OR (95%CI)	p
Subgroup analysis I: Age<60	Normal BP	Ref		Ref	
	Elevated BP	1.176 (0.820-1.686)	0.377	1.539 (0.888-2.669)	0.124
	Stage 1 hypertension	1.388 (1.048-1.837)	0.022	3.200 (2.048-4.999)	<0.001
	Stage 2 hypertension	1.886 (1.403-2.535)	<0.001	12.486 (6.905-22.576)	<0.001
Subgroup analysis I: Age≥60	Normal BP	Ref		Ref	
	Elevated BP	1.384 (0.892-2.148)	0.147	3.806 (1.608-9.007)	0.002
	Stage 1 hypertension	1.593 (1.107-2.292)	0.012	5.261 (2.446-11.318)	<0.001
	Stage 2 hypertension	1.510 (1.039-2.194)	0.031	19.245 (7.168-51.668)	<0.001
Subgroup analysis II: Male	Normal BP	Ref	-	Ref	
	Elevated BP	1.178 (0.820-1.692)	0.375	3.449 (1.878-6.336)	<0.001
	Stage 1 hypertension	1.325 (0.991-1.772)	0.057	14.612 (8.635-24.727)	<0.001
	Stage 2 hypertension	1.429 (1.049-1.945)	0.023	67.329 (37.550-120.726)	<0.001
Subgroup analysis II: Female	Normal BP	Ref	-	Ref	
	Elevated BP	1.392 (0.903-2.148)	0.135	3.246 (2.380-4.426)	<0.001
	Stage 1 hypertension	1.591 (1.130-2.239)	0.008	4.902 (3.760-6.392)	<0.001
	Stage 2 hypertension	1.931 (1.350-2.762)	<0.001	12.288 (8.695-17.365)	<0.001
Subgroup analysis III: Without using antihypertensive drugs	Normal BP	Ref	-	Ref	
	Elevated BP	1.287 (0.960-1.726)	0.091	2.963 (2.229-3.938)	<0.001
	Stage 1 hypertension	1.359 (1.064-1.736)	0.014	6.110 (4.777-7.815)	<0.001
	Stage 2 hypertension	1.504 (1.149-1.967)	0.003	23.147 (16.768-31.952)	<0.001
Subgroup analysis III: Using of antihypertensive drugs	Normal BP	Ref	-	Ref	
	Elevated BP	0.846 (0.353-2.029)	0.708	3.229 (1.264-8.247)	0.014
	Stage 1 hypertension	1.265 (0.698-2.295)	0.439	4.631 (2.178-9.847)	<0.001
	Stage 2 hypertension	1.260 (0.696-2.281)	0.445	12.992 (5.921-28.505)	<0.001

*BP: blood pressure; HR: hazard ratio; OR: odds ratio; CI: confidence interval; ACC/AHA: American College of Cardiology/American Heart Association; ASCVD: atherosclerotic cardiovascular disease; CVD: cardiovascular disease. For CVD: adjusted age (not adjusted in subgroup analysis I), gender (not adjusted in subgroup analysis II), educational level, drinking, body mass index, diabetes, antihypertensive drugs (not adjusted in subgroup analysis III), triglycerides, low-density lipoprotein cholesterol, high-density lipoprotein cholesterol, and glucose. For ASCVD: adjusted age (not adjusted in subgroup analysis I), gender (not adjusted in subgroup analysis II), educational level, drinking, smoking, body mass index, diabetes, antihypertensive drugs (not adjusted in subgroup analysis III), triglycerides, low-density lipoprotein cholesterol, high-density lipoprotein cholesterol, and glucose.*

## Discussion

This cohort study used the data from the CHARLS database to observe that patients with stage 1/2 hypertension defined by the 2017 ACC/AHA hypertension guideline were associated with a higher risk of CVD compared with normal BP among middle-aged and elderly people in China. Additionally, there was a positive association between individuals with elevated BP, stage 1 hypertension, stage 2 hypertension, and 10-year higher ASCVD risk.

In November 2017, the ACC and the AHA released a clinical guideline for the prevention, detection, and management of hypertension.^18^ Unlike the 2003 Seventh Report of the Joint National Committee (JNC7) which defined hypertension as SBP≥140 mmHg or DBP≥90 mmHg in the general population,^19^ the 2017 ACC/AHA guidelines recommend using a lower BP threshold to diagnose hypertension.^20^ Recent evidence suggests that the 2017 ACC/AHA hypertension guideline substantially increased the prevalence of hypertension.^21,22^ The application of the 2017 ACC/AHA guidelines has been a topic of global concern, particularly the impact of BP levels on CVD risk. Up to now, several studies have been conducted to assess the association between BP levels defined by the 2017 ACC/AHA hypertension guideline and CVD incidence.^9-11^ However, these results have been inconsistent due to the population’s selection. A retrospective study including 15,508,537 participants in Koreans aged 20-39 years demonstrated that stage 1 hypertension is connected with a higher risk for CVD.^23^ A multi-provincial cohort study in China showed that stage 1 hypertension defined by the 2017 ACC/AHA hypertension guideline was not related to the risk of CVD for participants aged ≥60 years.^10^ Similarly, a cohort study from Northern China illustrated that stage 1 hypertension [hazard ratios=1.25, 95% CI: 1.11-1.40] had a higher risk of cardiovascular events compared with normal BP.^24^ In comparison, our study is the first conducted in China to investigate the relationship between BP levels and CVD/ASCVD risk in middle-aged and elderly people (≥45 years old) based on the CHARLS database.

In this present study, after adjusting confounding factors, stage 1/2 hypertension was correlated with an increased risk of 7-year CVD. These results indicated that SBP ≥130 mm Hg or DBP ≥80 mm Hg was considered to as a risk factor for long-term CVD in middle-aged and elderly people. However, according to the 2018 CHL BP guidelines, SBP≥140 mmHg and/or DBP≥90 mmHg is commonly used to define hypertension in the Chinese population. Hypertension has been recognized as a CVD risk factor. In other words, the BP classification under the 2017 ACC/AHA BP guidelines may apply to the Chinese population. More research is needed to verify the findings in the future. Previous research has demonstrated that assessing ASCVD risk is a crucial step in the management of CVD prevention. This study suggests a positive correlation between elevated BP and increased risk of 10-year high ASCVD. Therefore, it is important to remain vigilant for the occurrence of high-risk ASCVD in patients with higher BP (SBP> 120mm Hg or DBP>80 mm Hg).

A prospective cohort study from China showed that stage 1 hypertension was associated with an increased risk of stroke in rural women aged ≥45, and stage 2 hypertension was associated with a significantly increased risk of stroke in women over 35 years of age compared with normal BP.^25^ In this study, we found that stage 1/2 hypertension exerted a significant impact on long-term CVD risk in both Chinese males and females.

Our study has several strengths. We used a CHARLS database which was a nationally representative sample of middle- and older-age Chinese adults, and the results might have broad generalizability in China. Additionally, BP was measured objectively and not self-reported in the present study. Nevertheless, this study also has several limitations. Firstly, since all data in this study was derived from the CHARLS database, the diagnosis of CVD and diabetes was based on the participants’ self-reports, which may underestimate the actual incidence of CVD and diabetes. Secondly, although we have adjusted several covariates that might confound the relationship between CVD risk and BP levels among middle-aged and elderly people, some confounders, such as laboratory indicators, living habits, physical activity, and family history of hypertension were not captured in this study. More prospective studies need to be performed in the future to explore this association between CVD risk and BP levels defined by the 2017 ACC/AHA hypertension guideline. Thirdly, the result of this study was based on a population of middle-aged and older Chinese adults, hence our findings may not apply to populations in other countries.

## Conclusion

In conclusion, this study indicated that the BP classification under the 2017 ACC/AHA BP guidelines may apply to the Chinese population. When SBP ≥130 mm Hg or DBP ≥80 mm Hg, middle-aged and elderly people may have a higher risk of CVD. Furthermore, heightened attention should be given to middle-aged and elderly individuals with elevated BP (SBP >120 mm Hg or DBP >80 mm Hg) due to their potential high risk of ASCVD.
